# Diffuse aspiration bronchiolitis: analysis of 20 consecutive patients*

**DOI:** 10.1590/S1806-37132015000004516

**Published:** 2015

**Authors:** Xiaowen Hu, Eunhee Suh Yi, Jay Hoon Ryu

**Affiliations:** Anhui Medical University, Anhui Provincial Hospital, Hefei, China. Division of Respiratory Medicine, Anhui Provincial Hospital, Anhui Medical University, Hefei, China; Mayo Clinic College of Medicine, Rochester, MN, USA. Mayo Clinic College of Medicine, Rochester, MN, USA; Mayo Clinic College of Medicine, Rochester, MN, USA. Mayo Clinic College of Medicine, Rochester, MN, USA

**Keywords:** Pneumonia, aspiration, Bronchiolitis, Gastroesophageal reflux, Lung diseases, interstitial

## Abstract

**OBJECTIVE:**

Aspiration can cause a variety of pulmonary syndromes, some of which are not well recognized. The objective of this study was to assess the demographic, clinical, radiological, and histopathological correlates of diffuse aspiration bronchiolitis (DAB), a bronchiolocentric disorder caused by recurrent aspiration.

**METHODS:**

This was a retrospective study of 20 consecutive patients with DAB seen at the Mayo Clinic in Rochester, Minnesota, between January 1, 1998 and June 30, 2014.

**RESULTS:**

The median age of the patients was 56.5 years (range, 22-76 years), and the male/female ratio was 2.3:1.0. In 18 patients, the diagnosis of DAB was based on the results of a lung biopsy; in the 2 remaining patients, it was based on clinical and radiological features, together with documented aspiration observed in a videofluoroscopic swallow study. In 19 patients (95%), we identified predisposing factors for aspiration, including gastroesophageal reflux disease (GERD), drug abuse, and dysphagia. Common presenting features included cough, sputum production, dyspnea, and fever. Twelve patients (60%) had a history of recurrent pneumonia. In all of the patients, chest CT revealed bilateral pulmonary infiltrates consisting of micronodules and tree-in-bud opacities. In the majority of patients, interventions aimed at preventing recurrent aspiration (e.g., anti-GERD therapies) led to improvement in the symptoms of DAB.

**CONCLUSIONS:**

Young to middle-aged subjects with recognizable predisposing factors for aspiration and who report a history of recurrent pneumonia are at increased risk for DAB. Although DAB is not well recognized, certain chest CT features are characteristic of the disorder.

## Introduction

Bronchiolitis, characterized by inflammation and fibrosis of the small airways, is associated with a variety of causes.^(^ 1 ^)^ In 1996, Matsue et al.^(^ 2 ^)^ described "diffuse aspiration bronchiolitis" (DAB) as a disorder characterized by chronic inflammation of the bronchioles due to recurrent aspiration. In that study, the diagnosis was made at autopsy in a cohort of mostly elderly bedridden subjects with neurological disorders and at obvious risk for aspiration. More recently, Barnes et al.^(^ 3 ^)^ reported the cases of four relatively young, healthy patients (41-59 years of age) with persistent bilateral pulmonary infiltrates, who were diagnosed with diffuse bronchiolar disease due to recurrent occult aspiration, the diagnosis being based on the evaluation of surgical lung biopsy specimens. Because of its atypical clinical and radiological presentation, DAB is not well recognized in clinical practice and often goes undiagnosed. The objective of the current study was to analyze patients with DAB treated at a tertiary referral center, in order to provide a more detailed description of the demographic, clinical, and radiological features, as well as the clinical course, of the disorder. 

## Methods

### Study subjects

Using a computer-assisted search of medical records, we identified 20 patients who were diagnosed with DAB at the Mayo Clinic in Rochester, Minnesota, between January 1, 1998 and June 30, 2014 (a period of more than 16 years). The current study was approved by the Mayo Clinic Institutional Review Board.

### Clinical data

Medical records were reviewed in detail, and data related to the following aspects were retrieved: age, gender, clinical presentation, comorbidities, predisposing factors for aspiration, radiological findings, histopathological features, diagnosis, treatment, and follow-up. The diagnosis of DAB was made by excluding other potential causes of pulmonary infiltrates and by applying the following diagnostic criteria^(^ 2 ^)^: respiratory symptoms (cough or dyspnea); features characteristic of bronchiolitis (i.e.*,* micronodules and tree-in-bud opacities) seen on chest CT scans; and histopathological (lung biopsy) evidence of foreign material or foreign-body giant cell reaction, associated with chronic, bronchiolocentric inflammation, or aspiration definitively demonstrated in a videofluoroscopic swallow study. Patients who were diagnosed with exogenous lipoid pneumonia were excluded. The predisposing factors and the diagnosis of aspiration-related pulmonary disease were assigned by consensus based on a review of all available medical records. 

### Statistical analyses

Continuous variables are presented as median and range (minimum, maximum), when appropriate, whereas categorical variables are presented as frequency and percentage. 

## Results

A total of 20 patients with DAB were identified; 14 (70%) were male (Table 1). The median age at diagnosis was 56.5 years (range, 22-76 years). The review of the medical records identified one or more predisposing factors for aspiration in 19 patients (95%), including gastroesophageal reflux disease (GERD) in seven (35%); a history of drug abuse in six (30%); dysphagia in five (25%); and GERD plus a history of drug abuse in one (5%). Six of the seven patients with a history of drug abuse had chronic opioid dependency. The median age was 45 years (range, 27-56 years) among the seven patients with GERD and 56.5 years (range, 22-76 years) among the six patients with a history of drug abuse, compared with 64 years (range, 57-69 years) in the five patients with dysphagia (p = 0.14). Four of the eight patients with GERD (including one of the patients with a history of chronic drug abuse) presented with active symptoms of GERD (i.e., heartburn and regurgitation). The four remaining patients with GERD were diagnosed with esophagitis by esophagogastroduodenoscopy. In one of those patients, there was spontaneous gastroesophageal reflux of barium into the cervical esophagus during an esophagram. Three of those four patients also underwent an esophageal motility study, and the lower esophageal sphincter pressure was found to be below normal in all three. Other comorbidities at the time of DAB diagnosis included malignancy, in three patients (esophageal cancer, thymoma, and chronic lymphocytic leukemia, respectively); depression, in two; chronic rhinosinusitis, in two; asthma, in two; chronic obstructive pulmonary disease, in one; obstructive sleep apnea, in one; cystic fibrosis, in one; and coronary artery disease, in one. 

**Table 1 -:** Demographic and clinical characteristics of patients with diffuse aspiration bronchiolitis.

Characteristic	N = 20
Gender, n (%)	
Male	14 (70)
Female	6 (30)
Age (years), median (range)	56.5 (22-76)
BMI* (kg/m^2^), median (range)	27.4 (17.3-51.8)
Main presentations, n (%)	
Cough	19 (95)
Sputum	16 (80)
Fever	16 (80)
Dyspnea	15 (75)
Hemoptysis	4 (20)
Recurrent pneumonia	12 (60)

BMI: body mass index.

* Data missing for one patient.

The most common presenting symptoms were cough (seen in 95% of the patients), sputum production (seen in 80%), fever (seen in 80%), and dyspnea (seen in 75%). One patient did not have active symptoms but was referred for evaluation of recurrent pneumonia. Twelve patients (60%) had a history of recurrent pneumonia. Among those 20 patients evaluated, choking episodes were reported by five (25%), although aspiration was suspected to be the cause of the lung disease in only three (15%). Overall, aspiration was unsuspected in 15 (75%) of the patients prior to the eventual diagnosis of DAB. 

In our study sample, the median body mass index (BMI) was 27.4 kg/m^2^ (range, 17.3-51.8 kg/m^2)^; six patients (30%) were obese (BMI ≥ 30 kg/m^2)^. Sixteen patients (80%) had a history of smoking. On examination, crackles were noted on lung auscultation in 14 patients (70%) and wheezing was heard in 2 (10%); digital clubbing was not observed. Pulmonary function test results were available for 11 patients, indicating an obstructive pattern in six (55%), a nonspecific pattern in one (9%), and normal function in the remaining four (36%). The DLCO was below normal in six of the 10 patients in whom it was measured. Arterial blood gas results were available for eight patients, one of whom was hypoxemic (PaO_2_ < 60 mmHg). 

**Figure 1 -:**
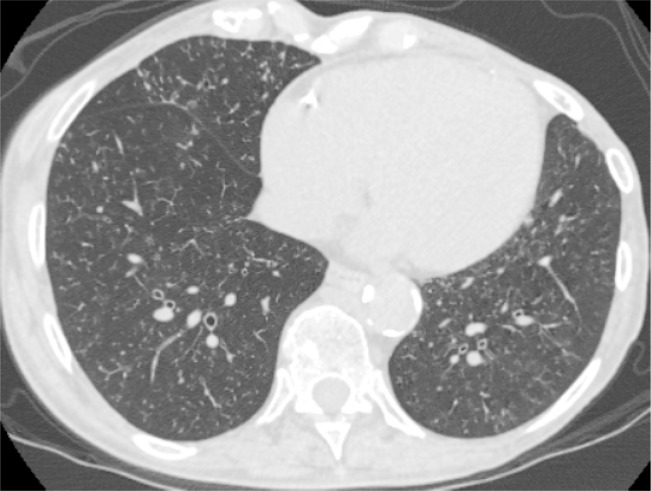
HRCT scan of the chest, showing diffuse micronodules and tree-in-bud opacities.

All of the patients underwent chest imaging studies, 17 undergoing chest X-ray plus CT of the chest and three undergoing chest CT only. Among the 17 patients for whom chest X-rays were available, bilateral involvement was observed in 15; the pattern was predominantly interstitial with patchy or diffuse distribution but was more micronodular in three patients. On the CT scans of the chest, bilateral distribution of parenchymal abnormalities was seen in 19 patients (95%) and posterior-dominant distribution of infiltrates was seen in only three (15%). One patient with unilateral lung involvement reported always sleeping on the ipsilateral side. Micronodules and tree-in-bud opacities consistent with bronchiolitis were seen on CT scans of the chest in all 20 patients (Figure 1); bronchiectasis was seen in seven patients (35%). 

**Figure 2 -:**
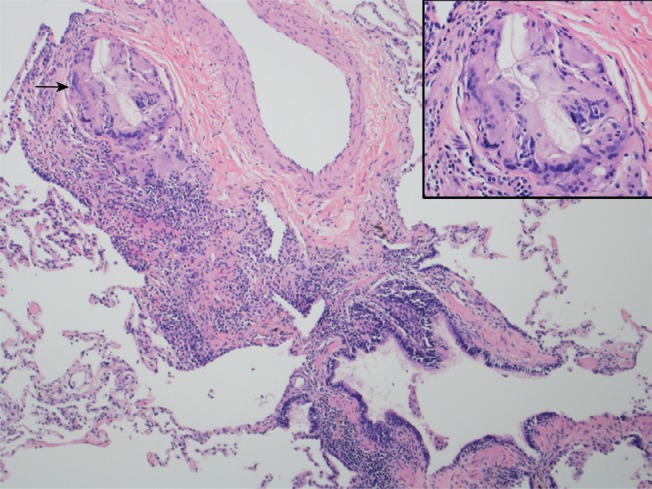
Photomicrograph of bronchiolar wall thickening (H&E staining; original magnification, ×100), showing chronic inflammation and a giant cell containing vegetable matter (arrow; inset magnification, ×400).

Among the 20 patients evaluated, the diagnosis of DAB was based on examination of the surgical lung biopsy specimen in 13 (65%); on examination of the transbronchial lung biopsy specimen in five (25%); and on the CT findings, together with gross aspiration demonstrated in a videofluoroscopic swallow study, in two (10%). Of the 13 patients diagnosed on the basis of the results of the examination of the surgical lung biopsy specimen (Figure 2), nine had previously undergone transbronchial lung biopsy that proved to be nondiagnostic. In eight of those nine, the biopsy specimens had been submitted to microbial culture; positive cultures were obtained in five patients-four testing positive for *Candida* spp., one testing positive for *Pseudomonas aeruginosa*, and one testing positive for *Staphylococcus aureus*. The five patients in whom the diagnosis of DAB was based on examination of the transbronchial lung biopsy specimen were among a total of 16 patients who had undergone transbronchial lung biopsy. Two remaining patients (10%) were diagnosed based on characteristic CT features and gross aspiration demonstrated on video swallow study. In 15 patients (75%), aspiration was not suspected prior to the establishment of histopathological evidence of DAB in transbronchial or surgical lung biopsy specimens (Figure 2). 

Seventeen patients were under pharmacological treatment for GERD, which included proton-pump inhibitors in 12, histamine type 2 receptor blockers in two, and a combination of the two therapies, together with lifestyle changes, in three. Of those 17 patients, 14 had been under pharmacological treatment for GERD before being diagnosed with DAB. In addition, empirical treatment included the use of prednisone in eight patients and bronchodilators in nine. There was no clear evidence that those medications provided any clinical benefit. Abstinence from drug abuse was achieved in six patients, leading to improvement in five. 

Three patients underwent surgical interventions to prevent recurrent aspiration: Nissen fundoplication in two; and Roux-en-Y gastric bypass in one. None of those three patients subsequently experienced recurrence of the aspiration. 

Of the 15 patients for whom follow-up information was available, 12 showed partial improvement or complete resolution of the symptoms and radiological abnormalities over a median follow-up period of 8 months (range, 1-74 months). Among the 20 patients evaluated, there were four deaths: two patients died of progressive cancer 5 and 8 months, respectively, after being diagnosed with DAB; one patient died of advanced cystic fibrosis; and one patient with opioid drug dependency died at home of unknown causes.

## Discussion

Since 1996, when Matsuse et al.^(^ 2 ^)^ proposed DAB as a new disease entity based on their autopsy study, there have been only a limited number of studies describing the clinical, radiological, and histopathological features associated with this disorder.^(^ 3 ^-^ 7 ^)^ The present study involved the largest sample of patients with DAB evaluated to date. 

Our results demonstrate that DAB is most commonly encountered in young to middle-aged subjects with identifiable predisposing factors and presenting with bilateral pulmonary infiltrates manifesting radiological features of bronchiolitis. It appears likely that many cases of DAB go undiagnosed, because the clinical and radiological features of DAB have not typically been associated with aspiration. Our data also show that GERD, a prevalent condition in the general population, was the most common predisposing factor for DAB, followed by drug abuse and dysphagia. Among our DAB patients, the median age was lower among those presenting with GERD or drug abuse than among those presenting with dysphagia. Dysphagia has been shown to be a common risk factor for aspiration-related pulmonary syndromes in prior studies, particularly among individuals with neurological disorders.^(^ 8 ^,^ 9 ^)^ In their study of elderly patients with DAB, Matsuse et al.^(^ 2 ^)^ found that half of those patients had oropharyngeal dysphagia. Similar to Barnes et al.,^(^ 3 ^)^ we identified GERD in nearly 40% of our DAB patients, suggesting that the risk for chronic occult aspiration is not limited to the elderly. 

The prevalence of GERD has been reported to be high in lung transplant recipients, and aspiration has been implicated as a contributor to the development of bronchiolitis obliterans syndrome in such patients.^(^ 10 ^,^ 11 ^)^ One recent study reported a reduced rate of decline in lung function after laparoscopic fundoplication in lung transplant recipients with GERD.^(^ 12 ^)^ In patients with idiopathic pulmonary fibrosis, GERD is highly prevalent and the use of acid-suppressive therapy has been associated with a longer survival, as well as with a slower rate of decline in pulmonary function. ^(^ 13 ^)^ Because GERD is quite common, additional exploration of occult aspiration-related pulmonary syndromes such as DAB seems warranted. 

Drug abuse has been considered a common predisposing factor for aspiration pneumonitis. ^(^ 14 ^,^ 15 ^)^ Similarly to GERD, chronic drug abuse, which affects relatively young subjects, likely predisposes to aspiration, possibly resulting in DAB.^(^ 16 ^)^ It is noteworthy that all of our patients with a history of drug abuse presented with chronic drug abuse, which has been associated with delayed gastric emptying.^(^ 17 ^)^

Cough, sputum production, dyspnea, and fever were the most common symptoms observed in our patients with DAB. A history of recurrent pneumonia was common in our patient sample (in 60% of patients) and could serve as a clue to the diagnosis of aspiration-related lung diseases. Recurrent pneumonia with persistent pulmonary symptoms that do not respond to antimicrobial therapy should alert clinicians to the possibility of recurrent aspiration.^(^ 2 ^,^ 7 ^,^ 18 ^)^

Although aspiration-related lung diseases are common, some forms are not well recognized. ^(^ 19 ^,^ 20 ^)^ Only 25% of our DAB patients were clinically suspected to have aspiration as the underlying cause of their lung disease. Bilateral lung involvement was noted on chest imaging studies for most of our patients with DAB. Although classic aspiration pneumonia has been associated with posterior-dominant distribution of infiltrates,^(^ 21 ^)^ only 15% of our DAB patients exhibited that feature. That might be explained by the small volume and recurrent nature of aspiration, which occurs mainly during sleep. Frequent changes in position during the night might lead to the diffuse distribution of aspirated material. In our patients with DAB, the chest CT findings (micronodules and tree-in-bud opacities) were characteristic of bronchiolitis, reflecting chronic bronchiolocentric inflammation caused by recurrent aspiration. Recognition of these radiological features could also serve as an important clue to the diagnosis of DAB.^(^ 3 ^)^

Management of patients with DAB focuses on prevention of recurrent aspiration by addressing the underlying risk factors, such as GERD and drug abuse. Our data demonstrate that this strategy led to improvement for the majority of DAB patients. Although these patients are unlikely to benefit from antimicrobial or corticosteroid therapy, there are virtually no data on the issue. Prior to being diagnosed with DAB (by surgical lung biopsy), one of our patients was treated with a 12-week course of prednisone for suspected hypersensitivity pneumonitis, and that treatment provided no clinical or radiological improvement. Optimal management strategies for patients with DAB have yet to be fully defined.

The present study has certain limitations. The major limitation is the retrospective design, which limited the analysis to clinical data available in medical records. Given the aforementioned impediments to the diagnosis of this disorder, it is likely that there were other patients with DAB who went undiagnosed at our institution and were not included in the study sample. 

We conclude that DAB is an under-recognized lung disease, that it is related to recurrent aspiration, and that it can be clinically occult. It is generally encountered in young to middle-aged adults with identifiable risk factors for aspiration, most commonly GERD. Radiological features consistent with bronchiolitis and a history of recurrent pneumonia are important clues to the diagnosis of DAB. 
